# ADAM12 promotes clear cell renal cell carcinoma progression and triggers EMT via EGFR/ERK signaling pathway

**DOI:** 10.1186/s12967-023-03913-1

**Published:** 2023-01-30

**Authors:** Jinming Xu, Yan Wang, Jiahao Jiang, Cong Yin, Bentao Shi

**Affiliations:** 1 Department of Urology, Shenzhen Second People’s Hospital/First Affiliated Hospital of Shenzhen University, Shenzhen, 518035 Guangdong China; 2 Shantou University Medical College, Shantou, 515041 Guangdong China; 3 Department of Urology, Peking University Shenzhen Hospital, Shenzhen, 518036 Guangdong China

**Keywords:** ADAM12, EGFR Pathway, EMT, Tumor Progression, Clear cell renal cell carcinoma

## Abstract

**Background:**

Clear cell renal cell carcinoma (ccRCC) is a major worldwide health problem due to its high prevalence and mortality rate. A disintegrin and metalloproteinase 12 (ADAM12) is aberrantly expressed in various cancers and plays an important role in tumor progression. However, its explicit effect and molecular mechanism in ccRCC remain unclear.

**Methods:**

We investigated the dysregulation of ADAM12 in ccRCC through public databases and bioinformatics analyses. The expression of ADAM12 was further verified in ccRCC tissues by RT-qPCR and immunohistochemistry (IHC). The relationship between ADAM12 expression and clinicopathological characteristics was analyzed statistically. The effects of ADAM12 on the proliferation, migration and invasion of ccRCC cells were examined by in vitro and in vivo experiments.

**Results:**

ADAM12 was significantly upregulated in ccRCC tissues and associated with poor prognosis in ccRCC patients. ADAM12 promoted ccRCC cell proliferation, migration and invasion in vitro and the growth of subcutaneous tumors in vivo. Knockdown of ADAM12 successfully suppressed its oncogenic function. Mechanistically, its overexpression induced epithelial-mesenchymal transition (EMT) by downregulating E-cadherin and upregulating N-cadherin and Snail. Moreover, ADAM12 participated in the epidermal growth factor receptor (EGFR) pathway and activated the downstream signal ERK1/2 by shedding the EGFR ligand, thereby upregulating target genes including c-Myc, enhancing cell survival and invasion ability, and promoting tumor progression, metastasis and the induction of EMT.

**Conclusions:**

High expression of ADAM12 induced EMT and promoted cell proliferation, migration, and invasion by activating the EGFR/ERK signaling pathway in ccRCC.

**Supplementary Information:**

The online version contains supplementary material available at 10.1186/s12967-023-03913-1.

## Background

Renal cell carcinoma (RCC) is a fatal genitourinary malignancy that accounts for approximately 5% of cancer cases in adult men and 3% in women [1, 2]. It was estimated that there were about 431 thousand incidences and 179 thousand deaths globally in 2020 (Global Cancer Observatory). RCC is a group of malignancies with diverse histologic types, specific cytogenetic properties, and different prognoses and therapeutic responses. It includes the most common subtype, clear cell renal cell carcinoma (ccRCC), which accounts for about 75%, papillary renal cell carcinoma (pRCC), chromophobe renal cell carcinoma (chRCC), and other less common subtypes [3]. Approximately 33 to 50% of patients have metastases by the time of detection [1], and nearly one-third of patients might experience recurrence or metastasis after nephrectomy. Patients with metastatic disease only have a median survival of 13 months [4]. Although the widespread application of imaging contributes to the early detection of tumors, further exploration of the molecular mechanisms underlying RCC development is urgently needed.

A disintegrin and metalloproteinases (ADAMs) are a multidomain and multifunctional family of type I transmembrane proteins [5] that are vital for regulating cell adhesion and mediating proteolysis of a variety of cell surface receptors and signal molecule extracellular domains. Substrates for ADAMs include Notch receptor and ligand, epidermal growth factor receptor (EGFR) ligand, interleukin-6 receptor (IL-6R), tumor necrosis factor (TNF) and its receptor, E-cadherin and CD44 [6]. Owing to their abnormal expression or dysregulation, ADAMs may lead to the initiation and progression of tumors [6].

The human ADAM12 gene is located on chromosome 10q26.3 and can alternatively splice to produce a long membrane-bound variant (ADAM12-L) and a short-secreted variant (ADAM12-S) [7]. As an active metalloprotease, ADAM12 is primarily responsible for the sheddases of EGF-like ligands, including heparin-binding EGF (HB-EGF), EGF, and betacellulin (BTC), thereby activating the EGFR pathway [8, 9]. In addition, studies have suggested that the expression of ADAM12 is increased in bladder [10], colorectal [11], gastric [12], lung [13], and breast cancers [6] and leads to poor prognosis. In renal cancer, Gao et al*.* found an upregulation of ADAM12 in ccRCC tissues and cells. Additionally, it was found to be significantly correlated with gender, TNM stage and clinical grade of patients [14]. However, the function of ADAM12 in ccRCC and its molecular mechanism have not been clarified. Here, we demonstrate the oncogenic role of ADAM12 in ccRCC, both in vivo and in vitro. In addition, we for the first time, demonstrate that ADAM12 can further induce EMT and promote tumor progression in ccRCC through the EGFR/ERK signaling pathway. Overall, the results of this study suggest that the functions of ADAM12 in modulating EGFR/ERK signaling and EMT contribute greatly to its role in ccRCC progression.

## Materials and methods

### ccRCC cell culture and tissue specimens

Human ccRCC cell lines (769-P, 786-O, Caki-1, Caki-2, ACHN) and the human proximal tubular epithelial cell line HK-2 were acquired from the American Type Culture Collection (ATCC). They were cultured at 37 °C and 5% CO_2_ in recommended culture media that contained 10% fetal bovine serum and 100 U/mL penicillin/streptomycin. Tissue samples were collected from patients diagnosed at the Department of Urology, Peking University Shenzhen Hospital, China. In total, 30 pairs of ccRCC tissues and adjacent normal kidney tissues were collected from ccRCC patients confirmed by pathology and before receiving any treatment. All experiments obtained ethical approval from the hospital’s Ethics Committee, and patients were informed of their specimen content, potential risks, and purposes of the study and signed written informed consent.

### Immunohistochemistry staining

Single spot tissue microarray (TMA) slides (150 cases of ccRCC, 30 cases of adjacent normal kidney tissues, HKidE180Su02, Shanghai Outdo Biotech Company, Shanghai, China) were prepared. The ADAM12 rabbit antibody (Proteintech, 1:3000) and the rabbit streptavidin–biotin detection system (Beijing Zhongshan Golden Bridge Biotechnology, Peking, China) were used for staining. According to the staining intensity (0 (negative), 0.5 + , 1 + , 2 + , and 3 +) and the staining positive rate (0–100%), two experienced pathologists evaluated the results. Then, the total score (0–300%) was calculated as the product of the staining intensity score and positive staining rate score. The low expression group had a score less than or equal to the median score, whereas the high expression group represented the opposite.

### RNA isolation and RTq-PCR

TRIzol reagent (Takara, Japan) was used to extract the total RNA from specimens. Then, for each sample, 1 µg of total RNA was reverse transcribed by the PrimeScript^™^ RT Reagent Kit with gDNA Eraser (Takara, Japan) and amplified by qPCR in a LightCycler 480 (Roche, USA) with the SYBR Premix Ex Taq^™^ II Kit (Takara, Japan). GAPDH was selected as the internal reference gene, and the relative expression was calculated by the 2^−ΔΔCt^ method. Primer sequences were synthesized by Sangon Biotech Company (Shanghai, China) and are listed below:

ADAM12-F: CGAGGGGTGAGCTTATGGAAC;

ADAM12-R: GCTTTCCCGTTGTAGTCGAATA;

GAPDH-F: CCACTCCTCCACCTTTGACG;

GAPDH-R: CTGGTGGTCCAGGGGTCTTA.

### Western blot

Protein extraction and western blot assays were conducted as previously described [15]. The primary antibodies used in this study were anti-ADAM12 (Proteintech, 1:1000), anti-E-cadherin, anti-N-cadherin, anti-Vimentin, anti-Snail, anti-ERK1/2, anti-phosphorylated-ERK1/2 (all from Cell Signaling Technology, 1:1000), anti-GAPDH (Abclonal, 1:7000), anti-c-Myc (Abcam, 1:1000), anti-EGFR (Abcam, 1:5000), and anti-phosphorylated-EGFR (Abcam, 1:3000). The secondary antibody used here was HRP-linked anti-IgG (Cell Signaling Technology, 1:2000).

### Viral infection

The human ADAM12 gene was amplified by PCR and cloned into a pHBLV-puro lentivirus vector. The ADAM12-targeting short hairpin RNA (shRNA) sequences were also cloned into pHBLV-puro, generated by HanBio (Shanghai, China). The shRNA sequences were CCACTATCTGCAAGACGGTACTGAT and ACCCATTCACCAGCCTCCATGAATT.

To establish a stable ccRCC cell line that could regulate the expression of ADAM12, 3 × 10^4^ cells per well were seeded in a 6-well plate and infected with the lentiviruses. After 48–72 h, the expression efficiency of GFP was preliminarily observed by fluorescence microscopy, and stable infections were selected with puromycin at 4 μg/ml twice.

### Drug treatment

Gefitinib (50 nM; Selleck, USA), an EGFR inhibitor, was dissolved in dimethyl sulfoxide (DMSO; Sigma‒Aldrich, Germany) and added to the corresponding medium. Meanwhile, the EGFR activator NSC228155 (100 μM, Selleck, USA) was dissolved in DMSO and used for subsequent rescue experiments. DMSO at 0.02% concentration was used as a control. Cells were harvested for 24 h, and then the corresponding inhibitor or activator was added for further western blot and functional assays.

### CCK8 assay

ccRCC cells were inoculated into 96-well plates at a concentration of 3 × 10^3^/ well with 100 µl culture medium. The plates were wrapped in tin foil and incubated for 5–6 h until the cells adhered to the wall. At 0 h, 24 h, 48 h and 72 h after transfection, 10 µl/well CCK-8 reagent was added under dark conditions. After 3 h of incubation, the culture plate was placed into a microplate reader, and the absorbance value (O.D. value) of each well was detected at 450 nm and then recorded.

### Colony formation assay

Five hundred cells were cultured in 6-well plates for 2 weeks. Then, the cells were fixed with 4% paraformaldehyde for 30 min and stained with 0.1% crystal violet solution for 30 min. After that, images of the colonies were taken after two washes with PBS. Finally, the crystal violet was eluted completely with 33.3% glacial acetic acid and transferred to 96-well plates. The absorbance of each well was detected with a microplate plate at 590 nm.

### Wound healing assay

Cells stably infected with lentivirus were cultured in 6-well plates. A linear scratch wound was created via a 10 µl pipette tip, and cell debris was removed by washing with PBS. Subsequently, the cells were supplemented with 2 ml of serum-free medium. The wound width was recorded by an inverted microscope every 24 h intervals, and the images were evaluated using ImageJ.

### Transwell assay

Cell invasion was examined by Transwell assay, where 200 µl serum-free medium containing 3 × 10^4^ cells was added to the upper chamber of Transwell (8 µm pore size, BD Biosciences, USA) coated with 50 µl Matrigel (BD Biosciences, USA). The lower chamber contained 500 µl of medium supplemented with 10% FBS. After 24 h of incubation at 37 °C, noninvaded cells were removed. Invaded cells were fixed with 4% paraformaldehyde, stained with 0.1% crystal violet, and examined under a microscope. After eluting crystal violet with 33.3% glacial acetic acid, the liquid was transferred to 96-well plates, where the absorbance value was examined by a microplate reader.

### RNA extraction and transcriptome sequencing

Total RNA was extracted as previously described [16]. After the assessment of RNA integrity, quality and quantity, the samples were transferred to Sangon Biotechnology (Shanghai) Co., Ltd. for library preparation and sequencing.

### Bioinformatics analysis

The limma package in R software (R version 4.0) was applied to compare gene expression profiles between shNC and shADAM12 cell samples, Vector and OE-ADAM12 cell samples. The cut‐off criteria for identifying differentially expressed genes (DEGs) were |log2-fold‐change (FC)|> 2 and adjusted P < 0.05. A Venn diagram was constructed to obtain the overlapping DEGs by FunRich (http://www.funrich.org) [17]. The RobustRankAggreg (RRA) R package was used to plot the distribution of DEGs on the heatmap [18]. DEG identification via Gene Ontology (GO) and Kyoto Encyclopedia of Genes and Genomes (KEGG) pathway enrichment analyses was conducted on the DAVID database (version 6.8, http://david.ncifcrf.gov), and their potential functional relevance was explored. Biological processes (BP), molecular functions (MF), and cell components (CC) were assessed by GO enrichment analysis, while pathways were enriched by KEGG pathway analysis. An adjusted P < 0.05 was regarded as statistically significant.

### In vivo xenograft model

The animal experiment protocol was approved by the Animal Ethics Committee of Shenzhen PKU-HKUST Medical Center. Twenty-four BALB/c nude mice (4–6 weeks) were split into 4 groups: shNC, shADAM12, Vector and OE-ADAM12. Transfected ACHN cells were resuspended in cold PBS buffer mixed with an equal volume of Matrigel. A mixture containing 3.5 × 10^6^ cells was subcutaneously injected into the axilla of each mouse. The tumor volume was measured and calculated in 3-day intervals by the following formula: tumor volume = (length × width^2^)/2. All mice were sacrificed one month after injection. The subcutaneous tumor specimens of the four groups were collected, weighed and photographed, and further immunohistochemistry staining experiments were performed.

### Statistics

GraphPad Prism 9 was utilized for statistical analysis, and data were presented as mean ± standard deviation (SD) with at least 3 repeats. Independent Student’s t test and one-way ANOVA were used for intergroup comparisons. Pearson’s chi-squared test or Fisher’s exact test was used to compare clinicopathological characteristics. Kaplan‒Meier survival analysis was performed for patient prognosis. P < 0.05 was considered statistically significant.

## Results

### ADAM12 was upregulated in ccRCC and correlated with poor prognosis

**Fig. 1:**
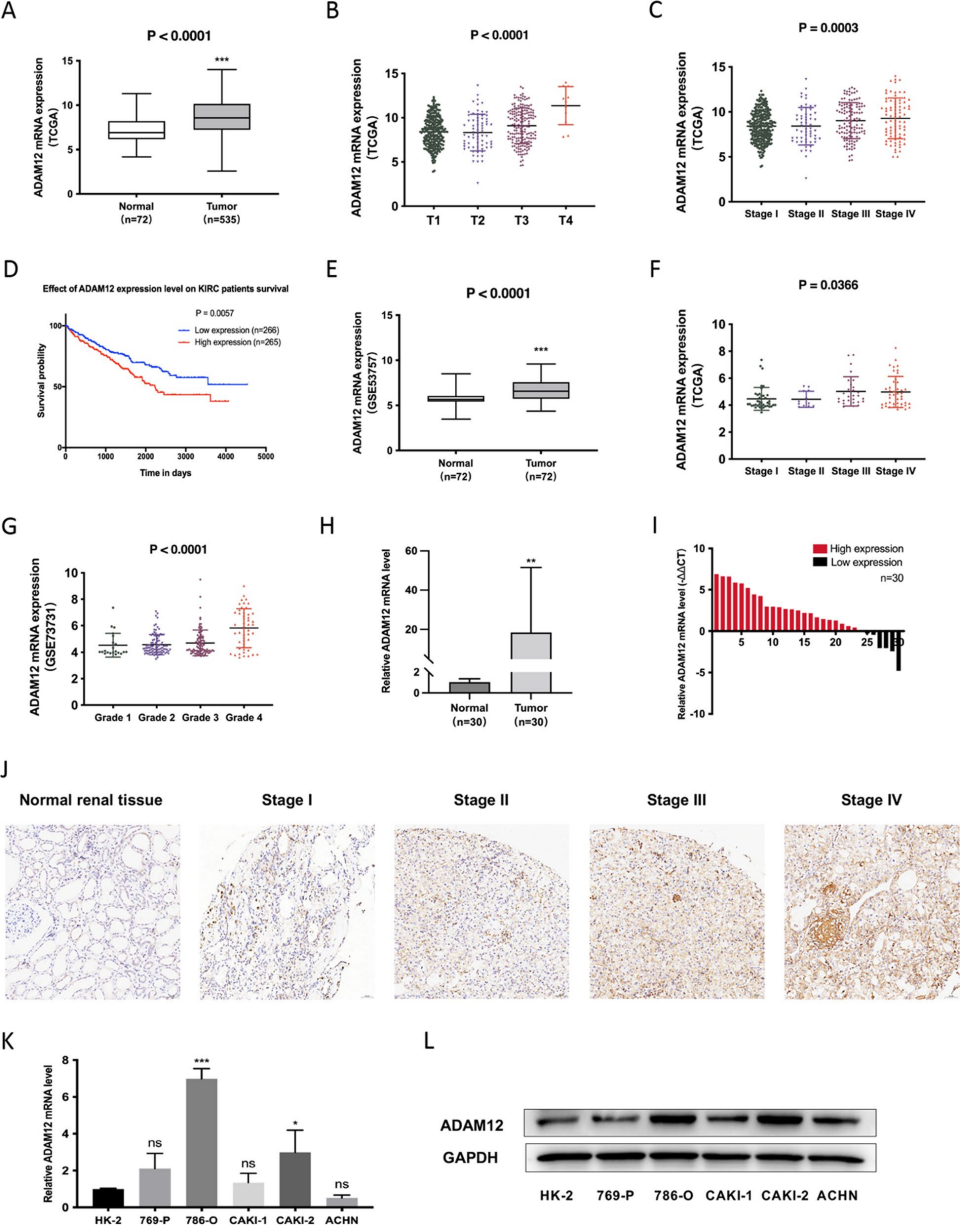
ADAM12 was upregulated in ccRCC tissues and cell lines. **A** The expression of ADAM12 in ccRCC tissues was significantly higher than that in adjacent normal kidney tissues according to TCGA. **B**, **C** ADAM12 expression was positively associated with T stage and tumor stage in ccRCC patients. **D** The expression of ADAM12 was negatively correlated with overall survival in ccRCC patients. **E** GSE53757 data set showed a significantly higher expression of ADAM12 in ccRCC tissues. **F**, **G** GSE73731 data set revealed that the expression of ADAM12 in ccRCC was associated with tumor stage and grade. **H**, **I** The relative expression of ADAM12 in 30 pairs of ccRCC tissues and adjacent normal kidney tissues. Statistical analysis revealed that ADAM12 was dramatically upregulated in 30 ccRCC tissues compared to normal tissues. **J** IHC assays showed ADAM12 protein expression in different tumor stages of ccRCC tissues and adjacent normal kidney tissues. Scale bar = 50 μm. **K**, **L** The expression of ADAM12 in ccRCC cell lines and HK-2 cells at both the transcriptional and translational levels. *P < 0.05, **P < 0.01, ***P < 0.001

To investigate the potential role of ADAM12 in ccRCC, we compared the expression profiles of ADAM12 between ccRCC tissues and adjacent normal kidney tissues with The Cancer Genome Atlas (TCGA) database and Gene Expression Omnibus (GEO) database. TCGA database revealed that ADAM12 was significantly upregulated in ccRCC tissues and was positively correlated with T stage and tumor stage in ccRCC patients (p < 0.001) (Fig. 1A–C). And its high expression indicated a poor prognosis for ccRCC patients (Fig. 1D). By analyzing the clinicopathological characteristics and clinical outcomes, we discovered that the dysregulation of ADAM12 in ccRCC was associated with age, gender, local invasion (T stage), lymph node involvement (N stage), and distant metastasis (M stage) (Table 1). Moreover, GSE53757 and GSE73731 data sets showed a significantly higher expression of ADAM12 in ccRCC tissues tightly associated with tumor stage and grade (p < 0.05) (Fig. 1E–G). Furthermore, we assessed ADAM12 expression in 30 pairs of ccRCC and adjacent normal kidney tissues and discovered that it was significantly upregulated in cancer tissues (Fig. 1H–I). These results were further confirmed by immunohistochemistry staining (p = 0.006) (Fig. 1J, Table 2). In addition, both the transcription and translation levels of ADAM12 detected in ccRCC cell lines suggested that ADAM12 had significantly higher expression in ccRCC cells than in the HK-2 cell line (Fig. 1K–L).

**Table 1:** Correlation between ADAM12 expression and clinicopathological characters of patients with clear cell renal cell carcinoma (TCGA)

Clinico- pathological variables	No. of cases	ADAM12 expression		χ^2^	p value
		Low	High		
All cases	535	267	268		
Gender	Male	157	192	9.723	0.0018**
	Female	110	76		
Age	$<$ 60	109	139	6.557	0.0104*
	$\ge$ 60	158	129		
Pathologic T	T1	147	128	9.143	0.0274*
	T2	39	31		
	T3	79	100		
	T4	2	9		
Pathologic N	N0	124	116	9.297	0.0096**
	N1	2	14		
	NX	141	138		
Pathologic M	M0	209	215	14.24	0.0008***
	M1	32	46		
	MX	25	6		
TNM stage	I	144	125	4.695	0.1955
	II	32	26		
	III	56	67		
	IV	35	47		
Survival	$<$ 5	181	200	3.612	0.0574
	$\ge$ 5	85	65		

^*^P < .05

^**^P < .01

^***^P < .001 was considered significant (Chi-square test or Fisher’s exact test)

**Table 2:** Differential expression of ADAM12 in cancer and adjacent tissues

Expression of ADAM12	No. of cases	Tissue type		Chi-square	p value
		Adjacent tissues	Cancer		
All cases	175				
Low	77	20	57	7.55	0.006**
High	98	10	88		

^**^P < .01 was considered significant

### ADAM12 promoted the proliferation of ccRCC cells in vitro

**Fig. 2:**
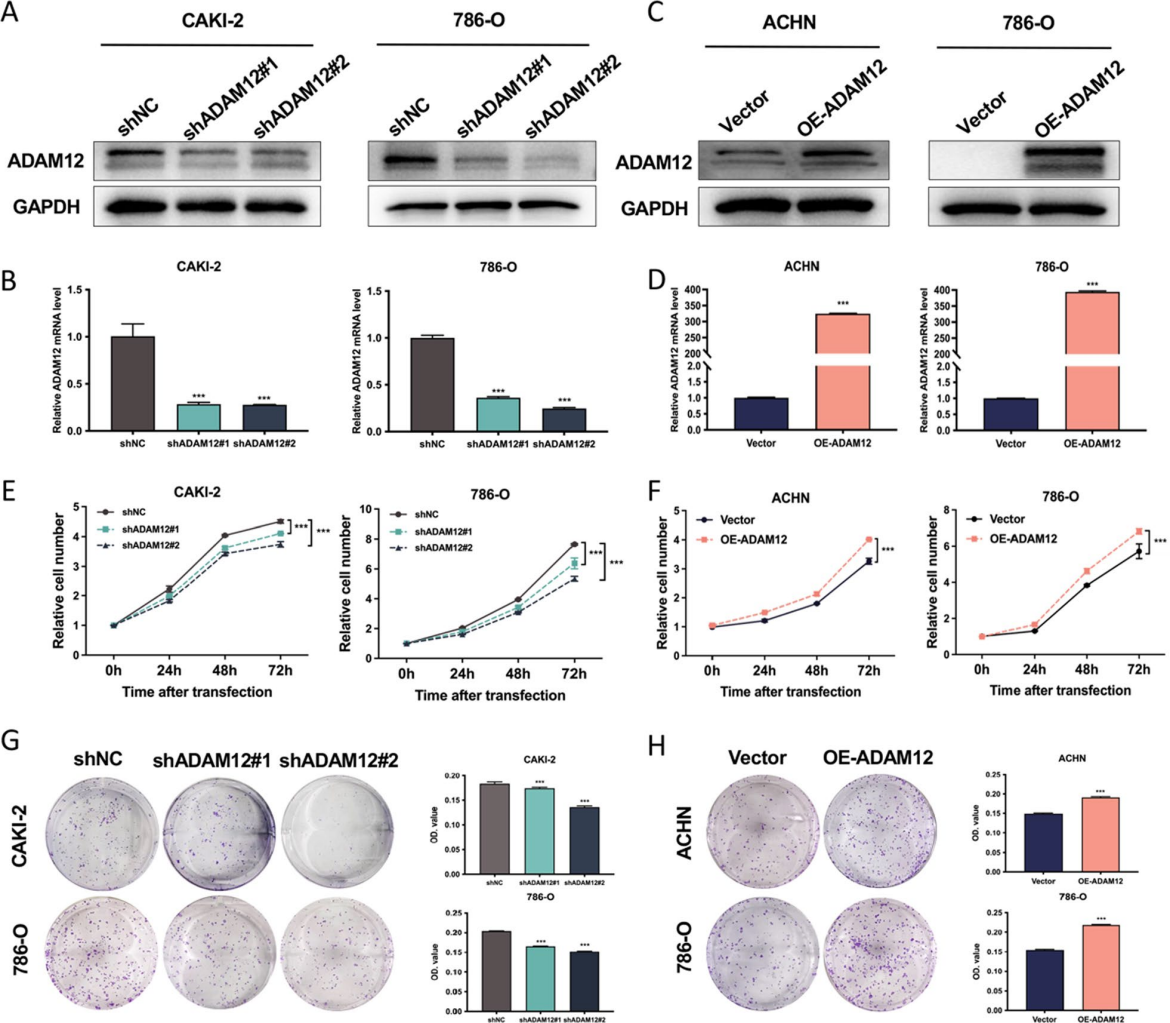
ADAM12 promoted the proliferation of ccRCC cells. **A**, **B** ADAM12 was significantly downregulated in CAKI-2 and 786-O cells at both the transcriptional and translational levels after infection with shADAM12 lentivirus. **C**, **D** ADAM12 was markedly overexpressed in ACHN and 786-O cells at both the transcriptional and translational levels after infection with OE-ADAM12 lentivirus. **E**–**F** CCK-8 assays showed the proliferation capacity of ccRCC cells infected with the indicated lentivirus. **G**, **H** Colony formation assay revealed the colony number of ccRCC cells infected with the indicated lentivirus

To explore the biological function of ADAM12 in ccRCC, ccRCC cell lines with stable ADAM12 knockdown or overexpression were established. The infection efficiencies were verified by RT‐qPCR and western blot experiments. Figure 2A, B confirmed the successful knockdown of ADAM12 in CAKI-2 and 786-O cells after infection with shADAM12 lentivirus at both the transcriptional and translational levels. In contrast, ADAM12 was markedly overexpressed in ACHN and 786-O cells when infected with OE-ADAM12 lentivirus (Fig. 2C, D). The CCK8 results revealed that downregulation of ADAM12 caused a remarkable inhibition in the viability of CAKI-2 and 786-O cells (Fig. 2E), whereas upregulation of ADAM12 had the opposite effect on proliferation (Fig. 2F). The colony formation assay suggested that its knockdown notably attenuated the colony formation capacity of CAKI-2 and 786-O cells. Conversely, its overexpression facilitated colony formation of ACHN and 786-O cells (Fig. 2G, H). These results confirmed that ADAM12 promoted the proliferation of ccRCC cells.

### ADAM12 facilitated migration and invasion and induced EMT in ccRCC cells

**Fig. 3:**
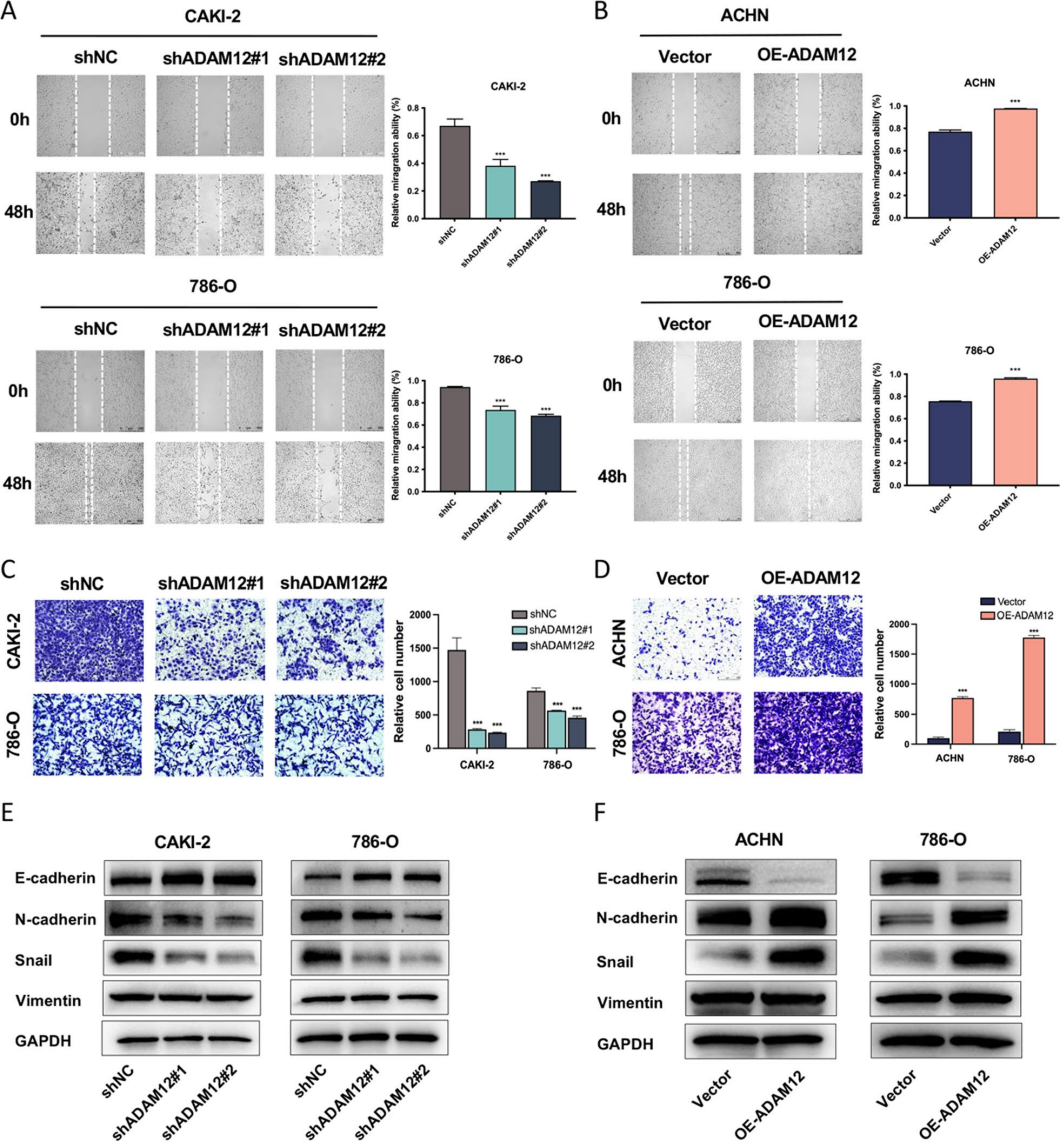
ADAM12 facilitated migration and invasion and induced EMT in ccRCC cells. **A**, **B** Wound healing assay showed the migration capacity of ccRCC cells infected with the indicated lentivirus. **C**, **D** Transwell assay revealed that the invasive capacity of ccRCC cells was obviously inhibited or strengthened after depletion or restoration of ADAM12 in ccRCC cells, respectively. **E** The effect of ADAM12 knockdown on EMT marker expression was assessed by western blotting. **F** The effect of ADAM12 overexpression on EMT marker expression in ACHN and CAKI-2 cells

Wound healing assays and transwell assays were conducted to investigate the effects of ADAM12 on the motility and invasiveness of ccRCC cells. The wound healing assay showed that silencing ADAM12 markedly inhibited the migration of CAKI-2 and 786-O cells, whereas restoring ADAM12 rescued the migration ability of ACHN and 786-O cells (Fig. 3A, B). Moreover, the transwell assay revealed that the invasive capacity of ccRCC cells was inhibited or strengthened after depletion or restoration of ADAM12 in ccRCC cells, respectively (Fig. 3C, D). To further examine whether EMT could be mediated by ADAM12, the expression of EMT transcription factors and related markers were analyzed by western blotting. In CAKI-2 and 786-O cells, after silencing ADAM12, the expression of E-cadherin was remarkably elevated, and the expression of N-cadherin and Snail were significantly reduced, while Vimentin was unaffected (Fig. 3E). We then overexpressed ADAM12 to explore whether it could reverse these changes. The results revealed that E-cadherin expression was downregulated, while N-cadherin and Snail expression were notably upregulated, leaving Vimentin intact (Fig. 3F). These changes further validated our results in the ADAM12 knockdown experiments and illustrated the role of ADAM12 in EMT induction.

### ADAM12 enhanced the EGFR/ERK signaling pathway in ccRCC

**Fig. 4:**
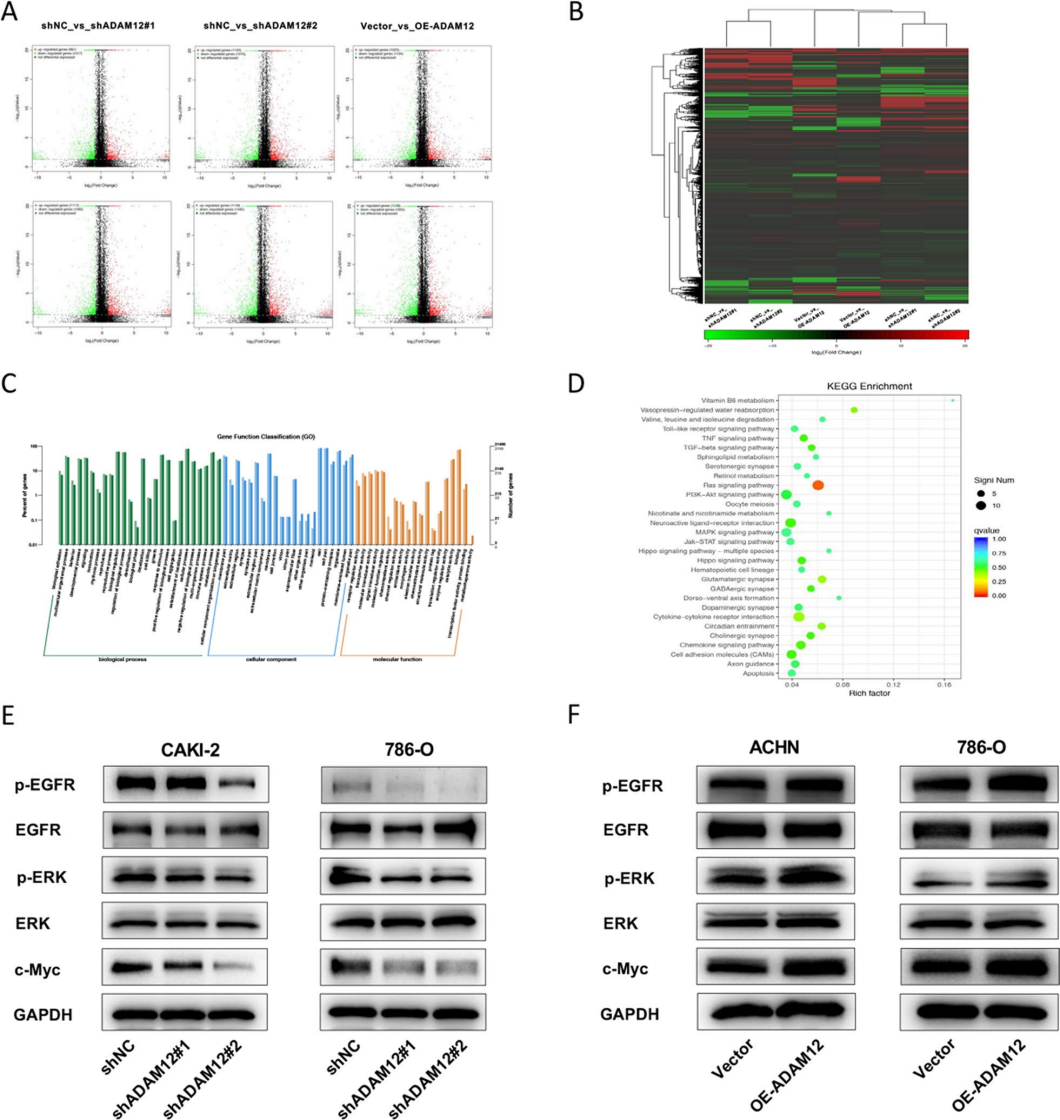
ADAM12 enhanced the EGFR/ERK signaling pathway in ccRCC. **A** Volcano plots of the distribution of DEGs by comparing shNC with shADAM12 cell samples and Vector with OE-ADAM12 cell samples. **B** The distribution of DEGs was plotted on the heatmap. **C**, **D** The biological functions of the integrated DEGs were explored by GO and KEGG enrichment analysis. **E**, **F** Western blot revealed the expression of EGFR/ERK and c-Myc proteins upon the knockdown and overexpression of ADAM12

To reveal the mechanism by which ADAM12 mediated the progression and EMT in ccRCC, we performed transcriptome sequencing and bioinformatics analysis. Volcano plots were generated for all DEGs by comparing shNC with shADAM12 cell samples and Vector with OE-ADAM12 cell samples (Fig. 4A). Notably, Venn software revealed that 17 overlapping DEGs were screened out (Additional file 1: Fig. S1, Additional file 1: Table S1). Through the RRA R package, the distribution of 17 overlapping DEGs was plotted on the heatmap (Fig. 4B). Then, GO and KEGG pathway enrichment analyses were conducted to explore the potential functional relevance among DEGs. The GO results suggested that DEGs were mainly involved in biological adhesion, multicellular organismal process, behavior and developmental process in BP; membrane part, extracellular matrix and region, and synapse in CC; and receptor regulator activity and transporter activity in MF (Fig. 4C). In addition, KEGG pathway enrichment analysis indicated that the Ras signaling pathway might be involved in ccRCC cells after ADAM12 stimulation (Fig. 4D). Moreover, previous studies reported that ADAM12-mediated shedding of EGFR ligands resulted in inducing the phosphorylation of EGFR and activating subsequent downstream signaling, including the Ras-MEK-ERK/MAPK, PI3K/AKT and JAK/STAT pathways [19, 20]. To further determine whether the EGFR/ERK pathway could be activated by ADAM12 in ccRCC cells, we conducted western blotting. The results revealed that silencing ADAM12 suppressed the phosphorylation levels of EGFR and ERK1/2 and downregulated c-Myc expression. However, overexpression of ADAM12 markedly promoted c-Myc expression and the phosphorylation of EGFR and ERK1/2 (Fig. 4E, F). These changes validated the role of ADAM12 in the EGFR/ERK signaling pathway.

### Silencing ADAM12 abolished the positive action of the EGFR activator on the proliferation, metastasis and EMT process of ccRCC cells

**Fig. 5:**
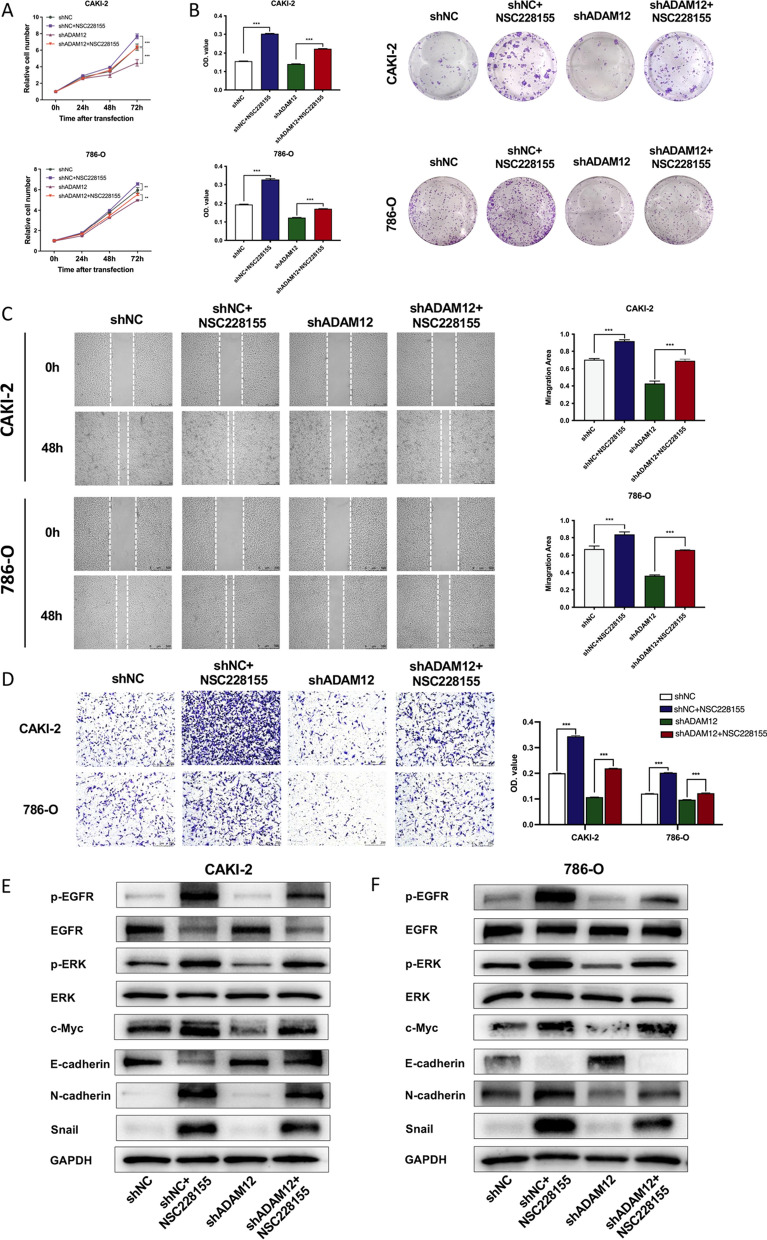
Silencing ADAM12 abolished the positive action of the EGFR activator on the proliferation, metastasis and EMT process of ccRCC cells. **A** CCK8 assay revealed the viability of CAKI-2 and 786-O cells treated with shADAM12 lentivirus and EGFR activator NSC228155. **B** Colony formation assays showed the colony number of CAKI-2 and 786-O cells treated with shADAM12 lentivirus and NSC228155. **C** The wound healing assay showed the migration capacity of CAKI-2 and 786-O cells treated with shADAM12 lentivirus and NSC228155. **D** Transwell assay indicated the invasion capacity of CAKI-2 and 786-O cells treated with shADAM12 lentivirus and NSC228155. **E**–**F** Western blot assay revealed the expression of the indicated proteins in CAKI-2 and 786-O cells treated with shADAM12 lentivirus and NSC228155

To examine whether its effect on EGFR/ERK signaling affected the progression and metastasis of ccRCC, we treated the cells with NSC228155, a specific EGFR agonist. The CCK8 assay revealed that the activation of EGFR dramatically elevated the viability of CAKI-2 and 786-O cells, and silencing ADAM12 counteracted this effect (Fig. 5A). Colony formation assays suggested that activating EGFR led to a remarkable increase in the colony number, while the knockdown of ADAM12 narrowed the change (Fig. 5B). Both wound healing and transwell assays indicated that the capacities of migration and invasion were improved after treatment with NSC228155. In addition, the depletion of ADAM12 reversed the promoting effect of NSC228155 on cell migration and invasion (Fig. 5C, D). Protein level analysis showed that the phosphorylation levels of EGFR and ERK1/2 rose significantly under NSC228155 treatment in CAKI-2 and 786-O cells. E-cadherin expression was markedly decreased, while c-Myc, N-cadherin and Snail expression were increased (Fig. 5E, F). However, this regulatory effect could be abolished by silencing ADAM12. Collectively, the EGFR phosphorylation activator reversed the inhibitory action of ADAM12 on proliferation, migration, invasion and EMT in ccRCC.

### EGFR blocking antagonized the effects of ADAM12 in ccRCC

**Fig. 6:**
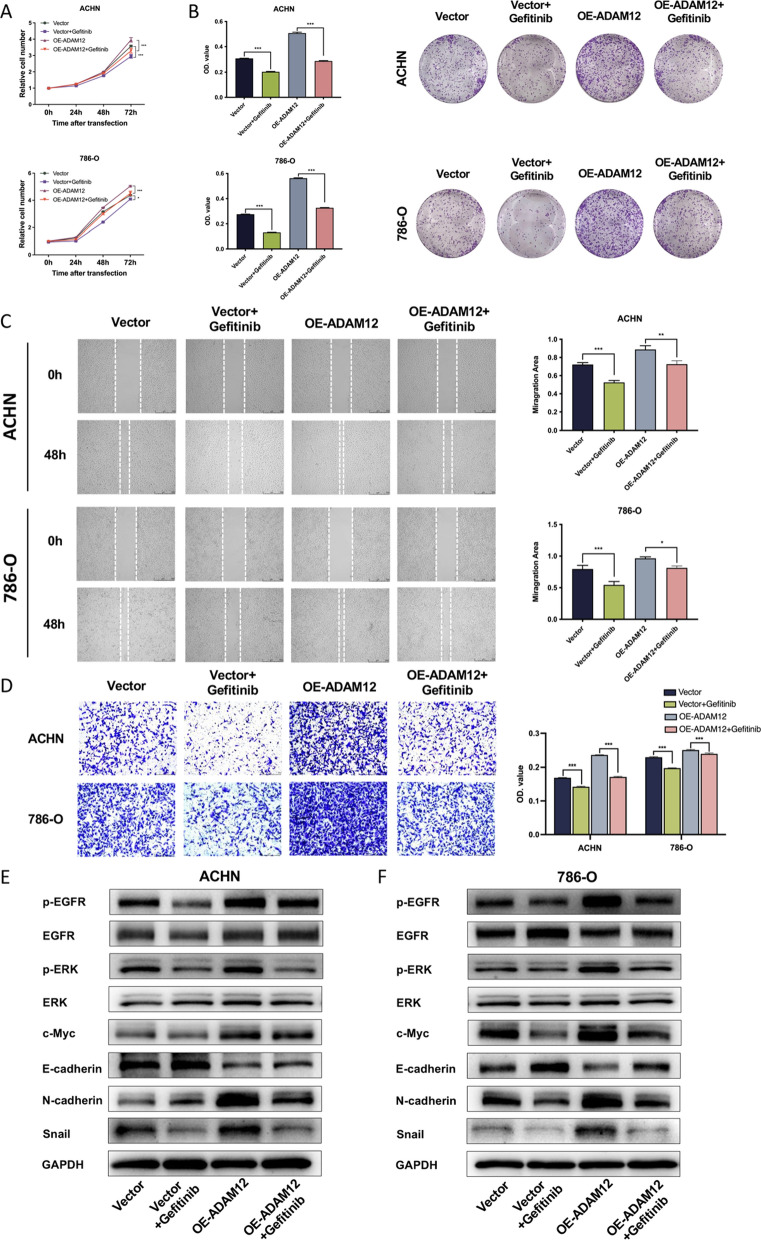
EGFR blocking antagonized the effects of ADAM12 in ccRCC. **A** CCK-8 assay revealed the viability of ACHN and 786-O cells treated with OE-ADAM12 lentivirus and the EGFR inhibitor gefitinib. **B** Colony formation assays showed the colony number of ACHN and 786-O cells treated with OE-ADAM12 lentivirus and gefitinib. **C** The wound healing assay showed the migration capacity of ACHN and 786-O cells treated with OE-ADAM12 lentivirus and gefitinib. **D** Transwell assay indicated the invasion capacity of ACHN and 786-O cells treated with OE-ADAM12 lentivirus and gefitinib. **E**, **F** Western blot assay revealed the expression of the indicated proteins in ACHN and 786-O cells treated with OE-ADAM12 lentivirus and gefitinib

To better clarify how ADAM12-induced proliferation, migration, invasion and EMT were regulated via the EGFR/ERK signaling pathway, ccRCC cells were treated with gefitinib, an EGFR phosphorylation inhibitor. Cell proliferation assays indicated that suppressing EGFR phosphorylation significantly reduced cell viability and cell growth in ACHN and 786-O cells. Overexpression of ADAM12 eliminated these inhibitory effects (Fig. 6A, B). Wound healing and transwell assays showed that gefitinib treatment dramatically decreased the number of migrated and invaded cells, while restoration of ADAM12 antagonized EGFR inhibition (Fig. 6C, D). In addition, western blot analysis showed that gefitinib significantly reduced the phosphorylation levels of EGFR and ERK1/2 and the expression levels of c-Myc, N-cadherin and Snail but increased E-cadherin expression (Fig. 6E, F). However, this inhibitory effect could be reversed by overexpression of ADAM12. These findings supported that the effects of ADAM12 on various ccRCC cell activities were achieved by phosphorylating EGFR.

### ADAM12 promoted the growth of ccRCC cells in vivo

**Fig. 7:**
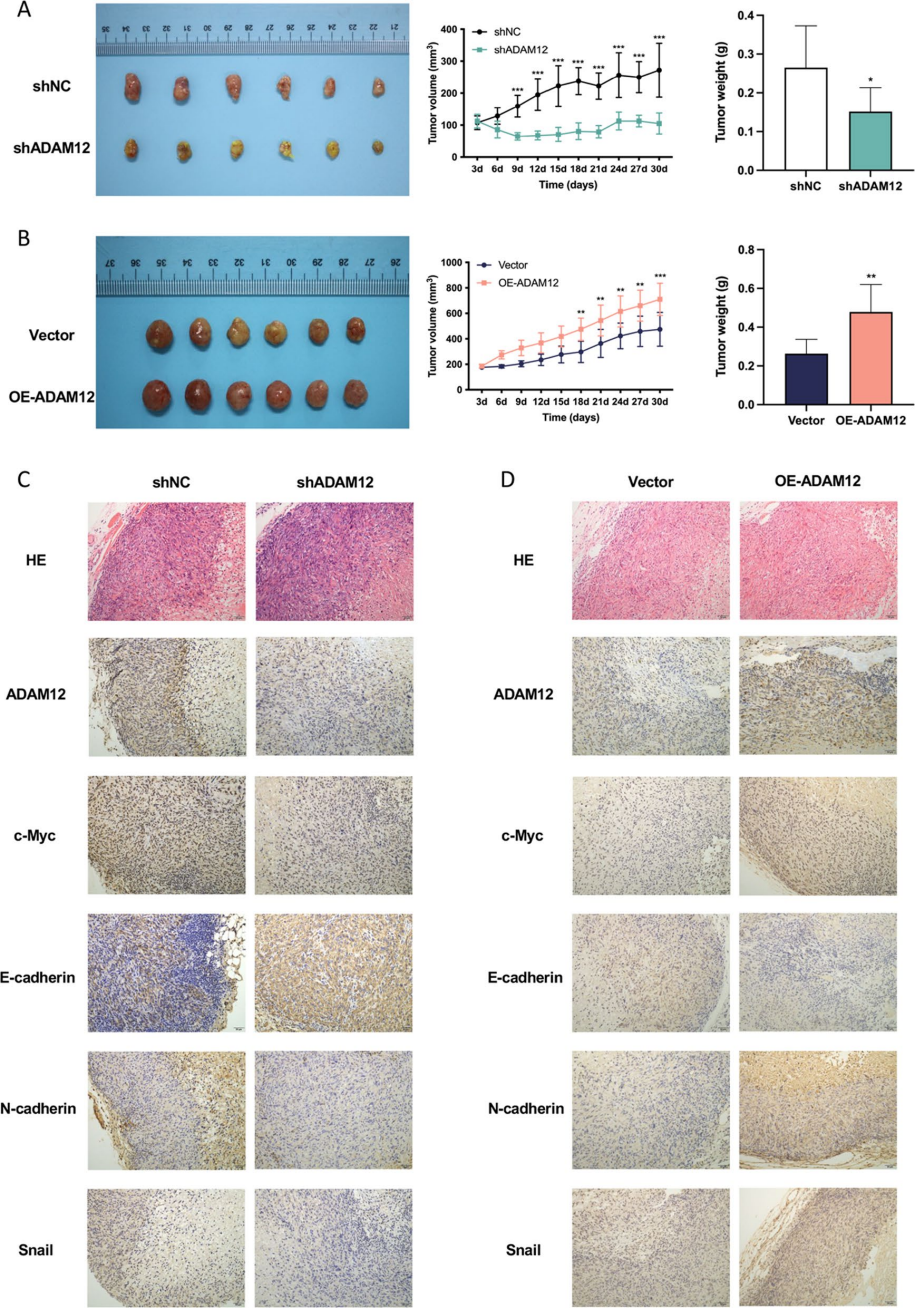
ADAM12 promoted the growth of ccRCC cells in vivo. **A**, **B** ACHN cells stably infected with shADAM12 or OE-ADAM12 were injected subcutaneously into the axilla of nude mice (n = 6 for each group) to create a xenograft tumor model. The tumor growth curves were documented according to the measurement of tumor volume every 3 days, and the tumor weight was measured. **C**, **D** Representative immunohistochemistry images of xenograft tumor tissues for HE, ADAM12, c-Myc, E-cadherin, N-cadherin and Snail staining

Finally, an in vivo model was established to gain more information on the role of ADAM12 in the growth of ccRCC. ACHN cells stably infected with shADAM12 or OE-ADAM12 were injected subcutaneously into the axilla of nude mice (n = 6 for each group). In the xenograft tumor model, ADAM12 knockdown significantly repressed the volume and weight of the tumors, whereas ADAM12 overexpression had the opposite effect (Fig. 7A, B). Moreover, the IHC assay demonstrated that the levels of ADAM12, c-Myc, N-cadherin and Snail drastically decreased after silencing ADAM12, whereas E-cadherin markedly increased, suggesting inhibition of tumor growth and the EMT process (Fig. 7C). The opposite effects occurred after overexpressing ADAM12 (Fig. 7D).

## Discussion

ADAM12, which belongs to the ADAM family, has unique characteristics, including extracellular metalloproteinase, cell-binding functions, and intracellular signal transduction capabilities [21]. It is worth noting that ADAM12, a secreted protein that can be detected in both blood and body fluids, is a good potential tumor biomarker. Previous evidence supported the oncogenic role of ADAM12 in multiple types of tumors. It promoted cell proliferation in glioblastoma through the stimulation of ectodomain shedding of proHB-EGF [22]. Moreover, ADAM12 significantly upregulated pro-angiogenic factors and increased endothelial cell recruitment in a STAT3-dependent manner to promote tumor angiogenesis in breast cancer [23]. Although Gao et al. predicted that ADAM12 might be a potential prognostic factor in ccRCC by bioinformatics analysis [14], both in vitro and in vivo experimental validations were lacking. This study could supplement this blank topic and elucidate the mechanism of ADAM12 in the development and progression of ccRCC.

In this study, we utilized the TCGA and GEO databases combined with our clinical specimens to evaluate the expression of ADAM12 in ccRCC and its correlation with clinicopathological factors and clinical outcomes. The data suggested that ADAM12 was highly expressed in most ccRCC tissues, mainly in the cytoplasm. Its expression was significantly correlated with gender, age, T stage, N stage and M stage. In addition, Kaplan‒Meier analysis showed that high expression of ADAM12 indicated poorer overall survival. Both our in vitro and in vivo assays demonstrated the oncogenic role of ADAM12 in ccRCC. We found that abnormally high ADAM12 expression promoted tumor growth and metastasis by enhancing cell proliferation, migration, and invasion. The positive effect of ADAM12 on tumor growth was further confirmed in a subcutaneous xenograft tumor mouse model. Nevertheless, ADAM12 knockdown significantly inhibited cell proliferation, clonogenicity, cell migration and invasion. Therefore, we hypothesized that ADAM12 might potentially be a therapeutic target for ccRCC treatment.

It was well-known that EMT mediated the response to various signaling factors, including embryonic development, fibrosis, wound healing, cancer metastasis, and proliferation [24]. EMT was typically activated in malignant tumors and acted as the initiation of tumor invasion and metastasis, triggering the separation of cancer cells from primary cancer and subsequently spreading to distant sites [25]. In this study, we observed that overexpression of ADAM12 induced the specific EMT transcription factor Snail. This could later reduce the expression of the epithelial marker E-cadherin and enhance the expression of the mesenchymal marker N-cadherin. Nevertheless, silencing ADAM12 had the opposite effects on EMT. These changes in all biomarkers indicated that ADAM12-mediated invasion and metastasis of ccRCC were correlated with the induction of EMT.

Previous studies reported that ADAM12 might mediate the release of soluble EGFR ligands, thereby activating EGFR [26, 27]. Notably, the phosphorylation of EGFR activated diverse intracellular processes, such as accelerating cell proliferation and invasion, suppressing apoptosis and stimulating tumor angiogenesis [28]. Moreover, EGFR was an upstream regulator of the Ras signaling pathway [29], and its activation was a major trigger for the progression of most carcinomas [30]. Since EMT could be modulated by multiple signaling pathways, including PTEN/Akt [31], Wnt/β-Catenin [32], PI3K/Akt [33], and MAPK [34], we first speculated whether ADAM12 induced EMT progression through the EGFR/RAS signaling pathway in ccRCC.

To validate these predictions, we conducted biological experiments. We found that ADAM12 overexpression markedly promoted EGFR and ERK1/2 phosphorylation and increased c-Myc expression. As an important target downstream of the ERK/MAPK pathway, a high level of c-Myc expression was tightly associated with cell proliferation and the induction of EMT in various carcinomas [35–38]. Cho et al*.* demonstrated that overexpression of c-Myc inactivated ERK-dependent GSK-3β and activated Snail, which could induce EMT in mammary epithelial cells [37]. In our study, we inhibited EGFR with gefitinib and discovered decreased phosphorylation levels of EGFR and ERK1/2. The transcription factors c-Myc and Snail and the mesenchymal marker N-cadherin were downregulated, whereas the epithelial marker E-cadherin was upregulated. These results suggested that inhibiting the EGFR/ERK pathway affected cell proliferation and EMT. However, overexpression of ADAM12 partially eliminated the effects, which was consistent with the evidence that ADAM12 was an upstream regulator of the EGFR/ERK pathway. These results proved that the oncogenic function of ADAM12 in cell proliferation and EMT was dependent on the activation of EGFR/ERK pathway. However, its two distinct isoforms in the regulation of EGFR/ERK signaling pathway in human cancers were unknown. The specific isoform of ADAM12 may provide greater help for the precisely targeted therapy of ccRCC in the future.

## Conclusion

Taking together, ADAM12 acts to facilitate the EGFR/ERK signaling pathway and enhance the function to activate c-Myc and EMT-related molecules such as E-cadherin, N-cadherin and Snail, leading to the induction of EMT and the promotion of ccRCC growth and metastasis. However, it remains unclear whether ADAM12-L or ADAM12-S plays a more vital role in ccRCC progression. All of the above will be further investigated in our future study.

## Supplementary Information

**Additional file 1. Table S1.** The relative expression of 17 overlapping DEGs via comparison between Vector and OE-ADAM12 group. **Figure S1.** Venn diagram representing the number of overlapping DEGs.

## Data Availability

The datasets used and/or analyzed during the current study are available from the corresponding author on reasonable request.

## Abbreviations

RCC: Renal cell carcinoma
ccRCC: Clear cell renal cell carcinoma
EGFR: Epidermal growth factor receptor
EMT: Epithelial-mesenchymal transition
IHC: Immunohistochemistry staining
RT‒qPCR: Reverse transcription-quantitative PCR
DEG: Differentially expressed gene
TCGA: The cancer genome atlas
GEO: Gene expression omnibus
RRA: Robust rank aggregation
GO: Gene ontology
KEGG: Kyoto encyclopedia of genes and genomes
