# Supplementary material for: ADAM12 promotes clear cell renal cell carcinoma progression and triggers EMT via EGFR/ERK signaling pathway
Source: J Transl Med. 2023 Jan 30;21:56. doi: 10.1186/s12967-023-03913-1 (PMC9885678; doi:10.1186/s12967-023-03913-1)

**Table S1.**The relative expression of 17 overlapping DEGs via comparison between Vector and OE-ADAM12 group.

| **Gene Name** | **Mean TPM (A2)** | **Mean TPM (A1)** | **log2 Fold Change** | **p Value** | **q Value** | **Result** |
| --- | --- | --- | --- | --- | --- | --- |
| AL132780.3 | 0.0001 | 22.28745 | -17.7659 | 7.41E-69 | 4.41E-67 | down |
| AC135050.2 | 0.0001 | 1.183266 | -13.5305 | 4.87E-15 | 5.99E-14 | down |
| LINC01010 | 0.0001 | 0.176674 | -10.7869 | 0.016403 | 0.041748 | down |
| MROH7-TTC4 | 0.011278 | 0.844779 | -6.22699 | 2.98E-27 | 6.63E-26 | down |
| AC005670.2 | 0.309421 | 6.539826 | -4.40161 | 1.41E-51 | 6.01E-50 | down |
| SPECC1L-ADORA2A | 0.19551 | 1.285241 | -2.71672 | 6.77E-27 | 1.49E-25 | down |
| AC005776.2 | 0.273339 | 1.04665 | -1.93702 | 2.02E-05 | 9.83E-05 | down |
| ANKRD65 | 0.081309 | 0.299898 | -1.88298 | 0.015716 | 0.040428 | down |
| AC008764.1 | 0.129019 | 0.428878 | -1.73298 | 0.002759 | 0.008844 | down |
| AC010531.1 | 1.531712 | 3.704173 | -1.27401 | 2.36E-05 | 0.000114 | down |
| SPTBN4 | 3.496872 | 1.020186 | 1.777233 | 1.39E-07 | 8.92E-07 | up |
| LDHD | 0.425672 | 0.096205 | 2.145558 | 0.001332 | 0.004631 | up |
| CLDN4 | 4.230521 | 0.828084 | 2.352986 | 1.55E-14 | 1.86E-13 | up |
| RAB4B-EGLN2 | 2.094442 | 0.180706 | 3.53485 | 9.98E-16 | 1.28E-14 | up |
| ENO1P4 | 7.823382 | 0.253228 | 4.949284 | 1.25E-69 | 7.57E-68 | up |
| AC007192.1 | 0.737216 | 0.0001 | 12.84787 | 1.29E-19 | 2.07E-18 | up |
| AL136295.1 | 4.689101 | 0.0001 | 15.51702 | 9.85E-53 | 4.30E-51 | up |

**Supplementary Figure 1.** Venn diagram representing the number of overlapping DEGs.


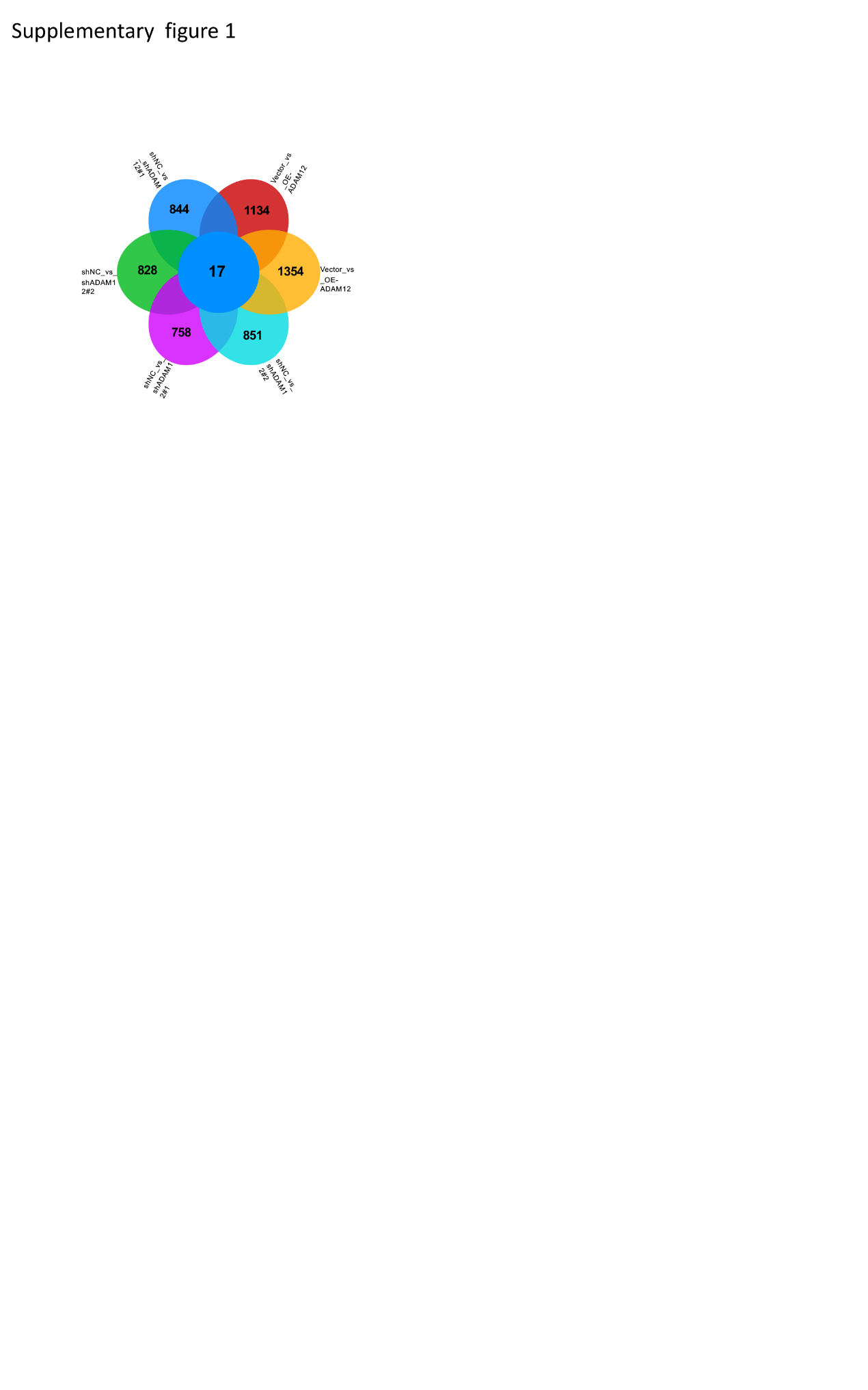

Supplement: Supplementary file 1 — Additional file 1. Table S1. The relative expression of 17 overlapping DEGs via comparison between Vector and OE-ADAM12 group. Figure S1. Venn diagram representing the number of overlapping DEGs. [file 12967_2023_3913_MOESM1_ESM.docx]
